# Exchange of Pb from Indian to Atlantic Ocean is driven by Agulhas current and atmospheric Pb input from South Africa

**DOI:** 10.1038/s41598-023-32613-5

**Published:** 2023-04-04

**Authors:** Saumik Samanta, Ryan Cloete, Subhra Prakash Dey, Jan-Lukas Menzel Barraqueta, Jean C. Loock, Jan-Olaf Meynecke, Jasper de Bie, Marcello Vichi, Alakendra N. Roychoudhury

**Affiliations:** 1 Department of Earth Sciences, Centre for Trace Metal and Experimental Biogeochemistry (TracEx), Stellenbosch University, Stellenbosch, 7600 South Africa; 2 Department of Oceanography, University of Cape Town, Rondebosch, 7701 South Africa; 3 Physical Oceanography Division, CSIR-National Institute of Oceanography, Dona Paula, Panaji, 403004 Goa India; 4 Coastal and Marine Research Centre, Griffith University, Gold Coast, QLD Australia

**Keywords:** Biogeochemistry, Environmental sciences, Ocean sciences

## Abstract

Using a spatiotemporal dataset of dissolved lead (dPb) from the subtropical oceans surrounding South Africa, this study quantifies the exchange of dPb between the Indian and Atlantic Oceans. Despite the absence of a major Pb source within the South Atlantic sector and the complete phase-out of leaded petroleum in Southern Africa, the ecologically important southeast Cape Basin shows an elevated surface dPb concentration (21–30 pmol kg^−1^). We estimated up to 90% of the measured dPb in surface waters of the Cape Basin was delivered from the Indian Ocean via the Agulhas Current (AC). Eddy dynamics and leakage at Agulhas retroflection result in an increased Pb flux from winter to summer, while a long-term (2008–2019) temporal change in dPb in the AC-derived water of Cape Basin was contemporaneous to a change in atmospheric Pb emissions from South Africa. The South African-origin atmospheric Pb, however, contributes first to the Agulhas waters in the West Indian Ocean, which is then transported to the South Atlantic, thereby regulating the dPb inventory of the Cape Basin. This indirect mechanism of Pb transfer emphasizes the importance of regulating Pb emissions from Southern Africa to protect rich fishing grounds associated with the Benguela marine ecosystem.

## Introduction

Lead (Pb) is introduced in the atmosphere through natural or anthropogenic sources^1–3^. Since the industrial revolution, the anthropogenic flux of Pb into the atmosphere has become the dominant source^4,5^, enhancing Pb input to the surface ocean. Therefore, modern-day dissolved Pb (dPb) concentrations in global oceans are generally higher compared to the natural oceanic background of 2.2 pmol kg^−1^^6^. In oceans, Pb is not known to play any role in biological growth and is primarily scavenged and advected as it is transported to the ocean interior^7–9^. Distribution of dPb in the water column is the result of one or a combination of the following processes such as historical Pb input in the surface waters, advection of subducted water, the vertical flux of Pb by sinking particulates, and legacy Pb signatures of mixing water masses^10,11^. Furthermore, natural and anthropogenic Pb entering into the ocean have distinct isotopic signatures (e.g., ^206^Pb/^207^Pb). Thus, dPb and its isotopic composition are often used to understand water mass mixing^7,12^, atmospheric Pb transport^13^, and the impact of anthropogenic activities in the oceans^14^.

Globally, the emissions of anthropogenic Pb to the environment have decreased since the 1980s, primarily in response to the phasing out of leaded petroleum since the 1970s^15^. For example, the atmospheric Pb emission from Europe, a major source of Pb in the North Atlantic and Arctic Oceans, and Mediterranean Sea^7,16,17^, dropped by an estimated 90% during the 1990s^18^. However, contemporary Pb emissions from some developing nations (e.g., India, China) rose owing to the increased use of other Pb-emitting raw materials^19–21^. Thus, being surrounded by developing nations and despite the phasing out of leaded petroleum^8,21^, measured dPb in the Northeast Indian Ocean show concentrations as high as ca. 90 pmol kg^−1^ (based on the collection between 2010 and 2016)^8,22,23^.

**Figure 1:**
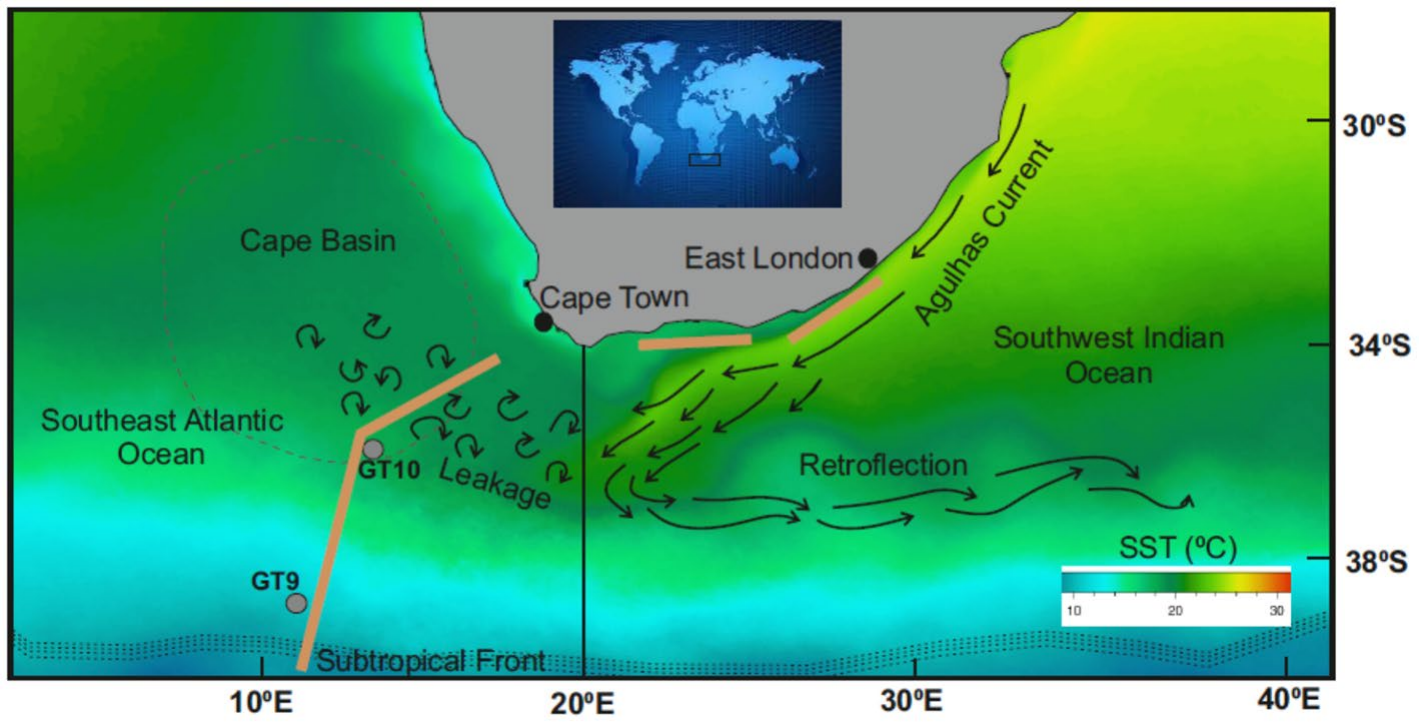
Sea Surface Temperature (SST) map of the study area showing the idealised pathway of Agulhas water and associated eddies that transfer seawater from the Indian Ocean to the South Atlantic Ocean. A zoomed-out map of the study area is shown within the black square in the globe. Surface water was sampled along the transects shown by light brown lines, and the grey circles (GT9 and GT10) indicate the location of CTD stations where vertical profiles were obtained. The black vertical line is the approximate boundary between the Indian Ocean and the Atlantic Ocean. Source of the SST data: https://oceancolor.gsfc.nasa.gov/.

The Cape Basin, located off the west coast of South Africa (Fig. 1), is a rich and biodiverse ecosystem supporting high productivity and industrial-scale fishing grounds^24^. Based on the European Union limit of toxic metals including Pb, selected predatory fishes and squids from the Cape Basin (observed between 2015 and 2017) are safe for human consumption^25^. Nevertheless, higher Pb inputs to the region may cause intense bioaccumulation of Pb in marine species, which negatively impacts the long-term sustainability of the ecosystem. From an oceanographic perspective, the southeast Cape Basin is characterised by local circulation driven by the fast-flowing western boundary Agulhas Current (AC)^26^. Approximately 25% of the AC-derived water (12.2 Sv), which originates in the Pb-rich Indian ocean sector, is known to leak into the Cape Basin^27^. Apart from the supply along with ocean water masses, dPb in the Cape Basin could also be supplied from the erosion of Southern African continental mass and aerosols generated in Southern Africa^28^. Between 2007 and 2008, South Africa phased out the use of leaded petroleum^29^, reducing Pb input into the surrounding environment and ocean. Nevertheless, as a coal-dependent developing nation, South Africa's use of other Pb-emitting raw materials could have increased Pb emissions in recent years and their effects on ocean water, as observed in South Asian countries^19,21^. Therefore, in order to understand the input and variability of dPb observed in the Cape Basin, it is essential to assess the sources and internal cycling of Pb.

By assessing the spatiotemporal distribution of dPb in the subtropical oceans around South Africa (Fig. 1), this research aims to identify potential sources of Pb in the Cape Basin and quantify the mixing of dPb caused by the interoceanic exchange in the upper water column. The dPb dataset was generated from samples collected from multiple oceanographic cruises between 2015 and 2019, and assessed in conjunction with previously published data^28,30,31^. The compiled dataset was also used to evaluate the near seasonal variation of the AC-derived water flux and associated dPb input in the Cape Basin. Based on the comparison between Pb emissions from South Africa and dPb concentrations in the Cape Basin, this study uncovers the importance of atmospheric Pb input from South Africa to the Indian Ocean-derived AC waters in influencing the dPb signature of the Atlantic waters of Cape Basin.

## Result and discussion

### Distribution of dPb in seawater

In the oceans surrounding South Africa (Fig. 1), dPb concentration in surface waters (< 5 m depth) ranged between 11 and 32 pmol kg^−1^ during August and November of 2019 (Supplementary table S1). The average dPb concentration was lower in the South Atlantic sector (average ± 1σ: 22 ± 5.8 pmol kg^−1^, n = 26) compared to the Indian Ocean sector (27 ± 2.8 pmol kg^−1^, n = 17). Within the South Atlantic sector, the elevated dPb concentrations were observed between 34 and 36.5°S, a sector within the southeast Cape Basin. Despite such spatial differences in dPb, in all post-2015 samples collected in this study, the measured dPb (Supplementary Table S1 and S2) in the southeast Cape Basin was approximately two to three times lower compared to previously published values (Sampling: 2008^31^). That is, the concentrations of dPb at a depth of 25 m at GT10 (Lat., Long.: − 36.3, 13.3; Fig. 1), a station where dPb was also monitored in the previous voyages^30,31^ (see “Methods”), declined from 49 to 21 pmol kg^−1^ between 2008 and 2019. Despite this decadal decrease, measured dPb in the Cape Basin in 2019 (26 ± 2.5 pmol kg^−1^, n = 9) was still higher compared to the seawaters sampled further south between 36.5 and 40°S (14 ± 2.4 pmol kg^−1^, n = 8) in the South Atlantic sector (Fig. 1), suggesting additional inputs of dPb to the Cape Basin.

Air mass trajectory models suggest that in the South Atlantic sector, aerosols are primarily derived from the remote marine (Pb-depleted) air masses originating from the southwest Southern Ocean and Patagonia^32,33^, while the Pb-enriched anthropogenic aerosols sourced from Southern Africa are transported east to the South Indian Ocean^34^. The resulting Pb laden aerosols from South Africa are deposited within the AC, flowing along the east coast of South Africa. Therefore, dPb and its variability in the Cape Basin, although supporting a nearby source, may be driven primarily by AC-driven oceanic processes (section "Contribution of Agulhas Current derived Pb to the South Atlantic"), which indirectly also transfer the aerosol contribution (section "Aerosol mediated Pb in the Cape Basin").

Based on Pb isotope data, a study identified three sources of dPb in the surface water of the Cape Basin: (1) the South Atlantic surface waters where Pb-bearing aerosols from South America are deposited, (2) South African shelf sediment in the form of particulate Pb, and (3) AC-derived Indian Ocean waters^28^. When measured in 2015 (Supplementary Fig. S1a), there was a linear correlation between total and dissolved Pb (r^2^ = 0.97, p < 0.05, n = 5), suggesting suspended particulates as a potential source of dPb. However, the estimated particulate Pb fraction (%), varied from 1 to 10% of the total Pb throughout the water column (Supplementary Fig. S1b). A previous study also reported less than 2 pmol kg^−1^ (< 10% of the dissolved load) particulate Pb concentrations in the Cape Basin^30^, which suggests suspended shelf sediments to be a weak source with only a minimal dPb contribution resulting from the exchange between particulate and dissolved Pb. That is, dPb distribution in the surface water of the Cape Basin may be primarily driven by the mixing with Indian Ocean waters.

### Contribution of Agulhas current derived Pb to the South Atlantic

**Figure 2:**
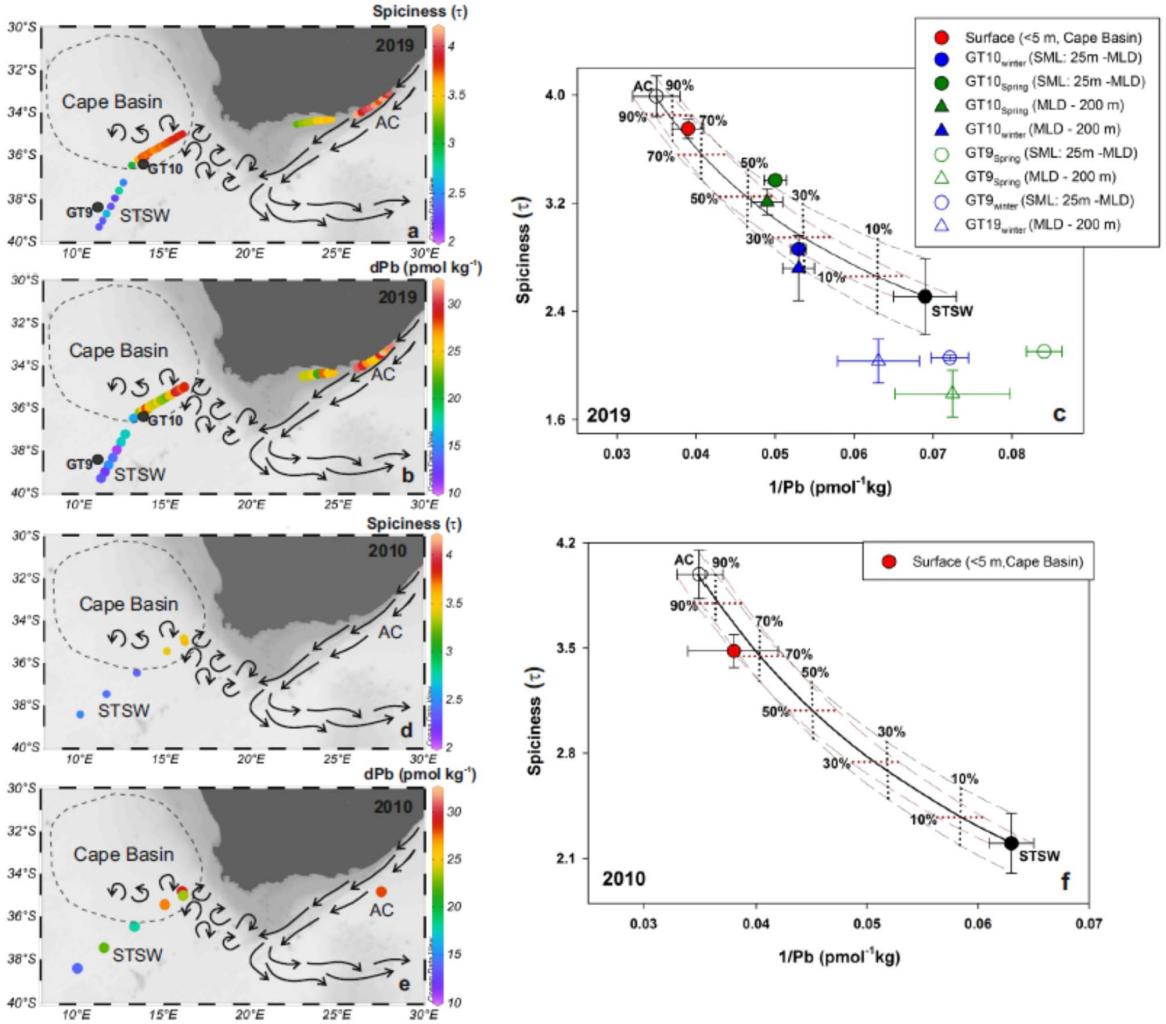
Distribution of τ (**a**) and dPb (**b**) in the Subtropical oceans around South Africa in 2019. (**c**) The mixing diagram of dPb was made using the data presented in the a and b panels. Distribution of τ (**d**) and dPb concentration (**e**) in 2010 from published literature^28,35^ and the corresponding mixing diagram (**f**). The arrows show the idealised circulation patterns. The end members (τ and Pb) for AC (open circle) and STSW (black-filled circle) in the mixing plots were calculated from the average and standard deviation of the samples collected in different oceanic regions representing the respective water masses shown in the left panels. The bold black curve in the mixing diagram (**c** and **f**) indicates the mixing between absolute values of the two end-members. The dashed curves also indicate the mixing considering the standard deviation associated with the absolute values of the end-members (black corresponds to τ and brown corresponds 1/Pb). The dotted lines represent the contribution of AC water constructed considering both the range of τ (black) and 1/Pb (brown). The τ value for the AC in 2010 (**f**) was drawn based on the 2019 dataset.

In order to assess the mixing of water masses with variable Pb signatures, a conventional mixing diagram based on dPb and Pb isotopic composition (^206^Pb/^207^Pb vs. 1/dPb) has been used in oceanography^7,35^. For instance, a plot of ^206^Pb/^207^Pb and 1/dPb suggests a linear mixing of two end-members (surface water masses) in the North Atlantic^7^. Similarly, assuming salinity as a conservative property, the plots of salinity versus Pb concentration and Pb isotopic compositions were used to show the mixing between two intermediate water masses along the isopycnal of tropical Atlantic (GEOTRACES GA06)^13^. A recent study also used a similar approach to determine the mixing of dPb in the surface water masses between South China Sea and Kuroshio core^36^. Surface waters, as in this study region; however, are often influenced by air-sea heat exchange, evaporation, and precipitation impacting in situ properties such as temperature, salinity, and density. Therefore, to overcome the non-conservative tendencies of in situ water-mass properties, we calculated spiciness (τ), a thermophysical property of seawater that determines potential density as a function of absolute salinity (S_A_), conservative temperature (T_c_), and pressure^37^ (see “Methods”). As potential density is a ‘potential property’, spiciness also possesses this potential property and is independent of the adiabatic and isohaline processes, supporting its use as an operational conservative tracer^37–39^. Based on spiciness (τ) values computed from the cruise transects, three different water masses of interest could be identified in the region (Fig. 2a). The AC waters along the east coast show the highest τ (3.99 ± 0.15) whereas the STSW waters between 36.5 and 40° S in the South Atlantic show the lowest τ (2.51 ± 0.28). In the southeast Cape Basin waters between 34 and 36.5° S, the τ values were higher (3.75 ± 0.07) compared to the rest of the South Atlantic waters, but still lower when compared to AC waters. dPb signatures also differ among these three water masses (Fig. 2b). The Cape Basin waters (26 ± 2.5 pmol kg^−1^) were enriched in dPb compared to the STSW (15 ± 2.4 pmol kg^−1^) and similar to the τ, the highest dPb concentrations were also found in the AC waters (29 ± 2.5 pmol kg^−1^).

In the Cape Basin, there is a lack of external source (due to the least input of atmospheric Pb) and minimum exchange between dissolved and particulate Pb that could change dPb concentration in the upper water column (section "Distribution of dPb in seawater"). Other factor that could make dPb to behave non-conservatively are the low residence time of dPb in surface seawater, riverine input to the coastal oceans, and the effect of evaporation-precipitation. Combined, all these factors have minimal quantitative impact on the dPb concentration in the study area (up to 0.02% of the measured dPb; see Supplementary Section ST1). Therefore, even though dPb is particle reactive and may behave non-conservatively in different reservoirs, it is possible to use dPb to develop the mixing diagram between two water masses (for example^7,13,35,36^).

In this study, the mixing diagram (τ versus 1/Pb) was constructed following the conventional mixing diagram of dPb (^206^Pb/^207^Pb versus 1/dPb) (see “Methods”). Two distinguished water masses (AC and STSW) formed the end members of the mixing diagram. Considering a variable Pb input over time and evolving dPb concentration in seawater, the proposed mixing diagram uses the dPb data of the end members and Cape Basin waters collected within the same year. The seawaters collected in 2019 shows that the surface waters (< 5 m) of Cape Basin lie on the mixing lines between the two end-member water masses (Fig. 2c). The Cape Basin waters were dominated by AC waters, accounting for 70 to 90% of the measured dPb as estimated from the mixing diagram. As a verification, a similar mixing diagram was constructed using estimated τ (based on published potential temperature: pT, practical salinity: S_p_, and pressure) and previously published dPb data from the South Atlantic and Indian Ocean sectors (year of collection: 2010–2011^28,35^; Fig. 2d–f). Due to the unavailability of pT and S_p_ data in the Indian Ocean sector in the observation from 2010^35^ (Fig. 2d), we use the τ value for the year 2019 to constrain the AC end member. In the case of dPb (n = 1; Fig. 2e), the standard deviation associated with the AC in the mixing plot (Fig. 2f) was calculated from the analytical precision of dPb, which is better than 5%. The result of the second mixing diagram also indicated AC-influenced water in the Cape Basin, supplying 70 and 85% of dPb from the Indian Ocean.

For additional verification of our approach, the results were compared with the results from a conventional mixing diagram based on 2010^28,35^ dataset of dPb and ^206^Pb/^207^Pb. Here also, the results show that the AC-influenced water in the Cape Basin was responsible for 70 to 85% of the measured dPb (Supplementary Fig. S2), consistent with the estimation using τ and dPb concentration (Fig. 2f).

The maximum depth where AC-derived water exists in the South Atlantic sector is 200 m^27^.Therefore, we further examined the mixing behaviour in the lower part of the surface mixed layer (SML: 25 m to mixed layer depth, MLD) of two CTD stations from the South Atlantic sector (GT9 and GT10; Fig. 1). The data-point GT9_spring_ (SML: 25–75 m, n = 3; Supplementary Table S2) deviates from the mixing line suggesting the available water mass (STSW) had no contribution from AC waters (Fig. 2c). In contrast, GT10_spring_ (SML: 25–45 m, n = 2) lies on the mixing line but was less dominated by the AC (45% contribution of the measured dPb) compared to the surface water (70–90% of the measured dPb). A similar fraction of AC-derived dPb was estimated at GT10 station for the water layer between the MLD and 200 m. Indeed, here as well the estimated contribution of AC-derived Pb to the Cape Basin, based on the SML (GT10) signal, was lower (45–50%) compared to that calculated from the surface water signal (70–90%). This variable response in the upper water column could simply be due to higher dPb concentration in the AC_surface_ than the average of AC_SML_ samples. High dPb in AC surface waters can result from the deposition of Pb-bearing aerosols, generated in Southern Africa, that travel east^34^ before being deposited within the AC waters or in the source waters of AC in the Indian Ocean (see section "Aerosol mediated Pb in the Cape Basin"). The low AC_SML_ value due to dilution on mixing with deeper waters is logical but could not be confirmed because, to the best of our knowledge, published data on vertical dPb distribution in AC (off the South African Coast) is lacking.

### Seasonal variation of AC-derived Pb to the Cape Basin

On including the GT10_winter_(SML: 25 to 142 m, n = 5) signal on the mixing plot (Fig. 2c) we observe that the data point lies on the mixing line, but is least influenced by AC waters (ca. 30% AC-derived dPb). This indicates that there is a seasonal variation in the AC-derived dPb input in the Cape Basin. The interoceanic exchange is made possible by Agulhas leakage in the form of eddies, shedding, and filamentation that occur at the Agulhas Retroflection^27^ (Fig. 1). The strength of Agulhas leakage, and thus the associated chemical flux, is known to vary due to the seasonal migration of the STF and the strength of the AC itself^40–42^. Although the seasonal movements of the STF to the south in summer and to the north in winter have been reported^43^, we found a negligible position shift between winter (between 37.15 and 41.78° S) and spring of 2019 (between 38.90 and 41.17° S) (see “Methods”). The monthly average sea surface temperature map (MODIS-Terra SST, 11 µ daytime; https://oceancolor.gsfc.nasa.gov/) shows an insignificant change in the SST at GT10 station between two sampling seasons (Supplementary Fig. S3). Thus, the observed decrease in the winter contribution of dPb to the Cape Basin is likely associated with the strength of the AC. Based on the observation from moorings across the AC, water volume transport revealed that the AC is over 25% stronger in austral summer than in winter^42^. Seasonal anomalies of the boundary layer transport additionally show an increasing AC-derived flow from winter to summer^41,44^, which would support the observed seasonal variability in the dPb flux to the Cape Basin.

**Figure 3:**
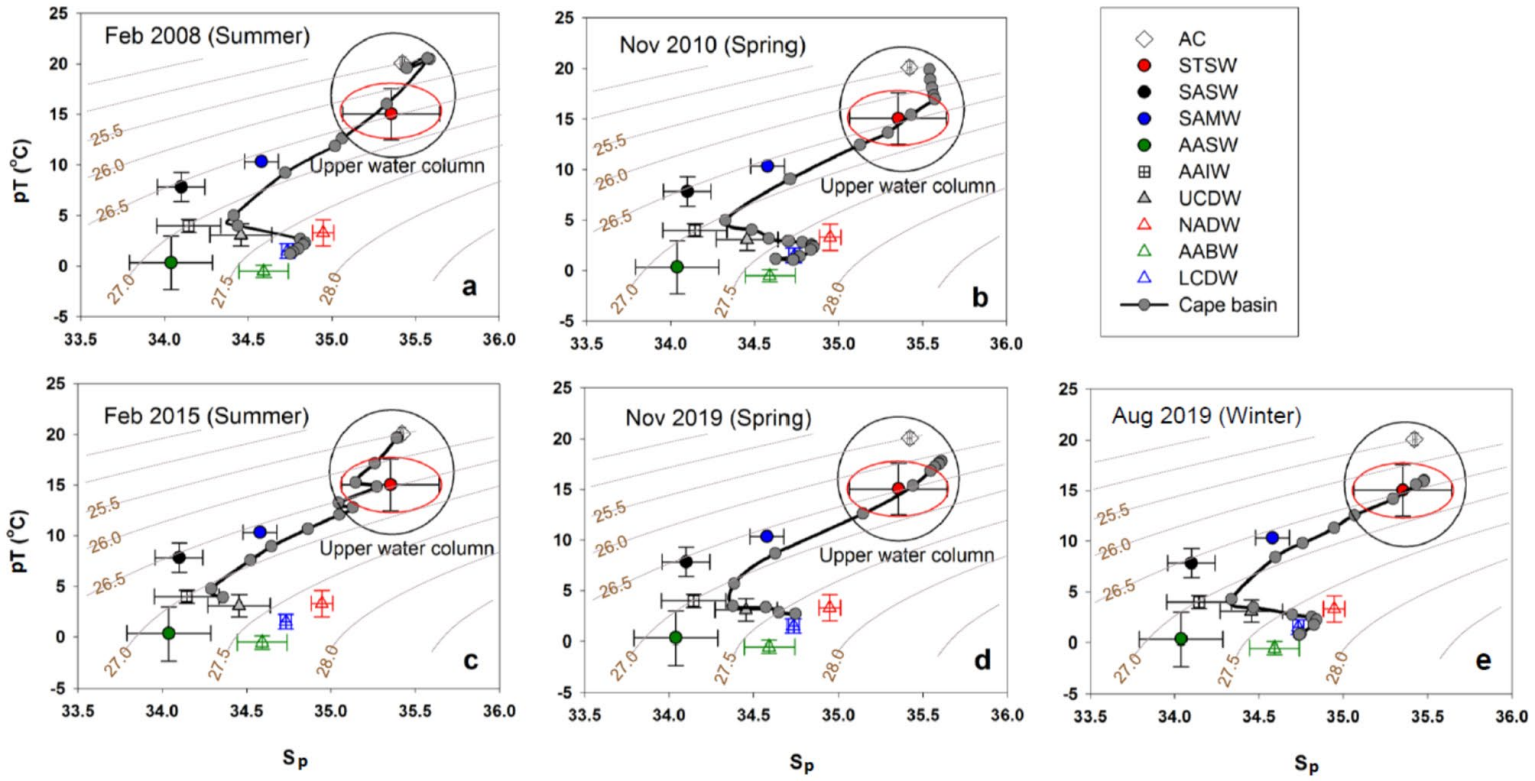
pT and Sp properties of the water column in the Cape Basin during (**a**) Feb 2008^31^, (**b**) Nov 2010^30^, (**c**) Feb 2015, (**d**) Nov 2019, and (**e**) Aug 2019. The data points that fall close to the STSW (red circle) and AC (open diamond, error bars within the symbol) correspond to the upper water column (ca. up to 400 m depth), located inside the black circle. The pT and Sp of identified water masses in the region are given in the “Methods” section. The red oval exhibits the boundary of STSW-rich water.

To further assess the seasonality, we constructed pT versus S_p_ plots using available temporal data collected in the water column within the southeast Cape Basin in summer (Feb 2008^31^ and Feb 2015), spring (Nov 2010^30^ and Nov 2019), and winter (Aug 2019, Fig. 3). The upper water column in the Cape Basin generally comprises STSW and overlying AC-derived water during summer and spring. During summer (Feb 2008 and Feb 2015), the pT-S_p_ properties of the upper waters, particularly from the SML, overlapped with AC waters (Fig. 3a, c); while in other seasons, its absence is noted (Fig. 3b, d, e). More specifically, in spring (Nov 2010 and Nov 2019), the SML waters were located between the AC and STSW suggesting the moderate influence of AC water, while they fall within the error bars of STSW in winter (Aug 2019) indicating little influence of AC water. Consequently, the contribution of AC-derived water was maximum in summer, intermediate in spring, and minimum in winter, agreeing with our estimation of AC-derived Pb input to the Cape Basin.

**Figure 4:**
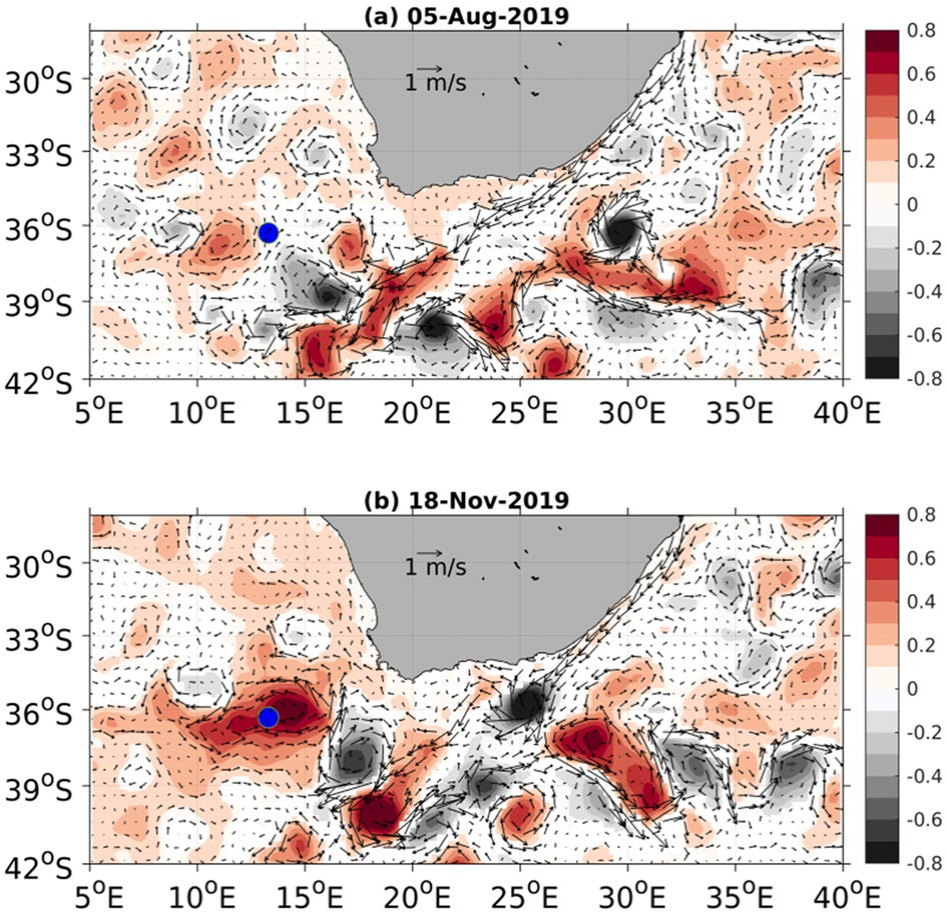
SLA anomalies (in m) between winter (**a**) and spring (**b**), 2019. The overlaying vectors represent the geostrophic currents (in m/s). The blue dot represents the GT10 station. (Data source: https://cds.climate.copernicus.eu/cdsapp#!/dataset/satellite-sea-level-global?tab=form).

The seasonality of AC-derived water and associated dPb flux, as discussed above (Figs. 2 and 3), have been confirmed using the Sea Level Anomaly (SLA). Given there is an interannual variation in the Agulhas Current leakage to the South Atlantic^45^, we evaluated the SLA between winter (5th august) and spring (18th November) in the year 2019, the date of sampling at GT10 (Fig. 4). The SSH anomalies show the existence of a strong anticyclonic eddy, the core of which was spatially associated with GT10 in spring. In contrast, eddy activity at GT10 during winter was weak. These observations are consistent with the increased contribution of AC-derived dPb to GT10 during spring (45%) as opposed to winter (30%).

### Decadal evolution dPb in the Cape Basin

**Figure 5:**
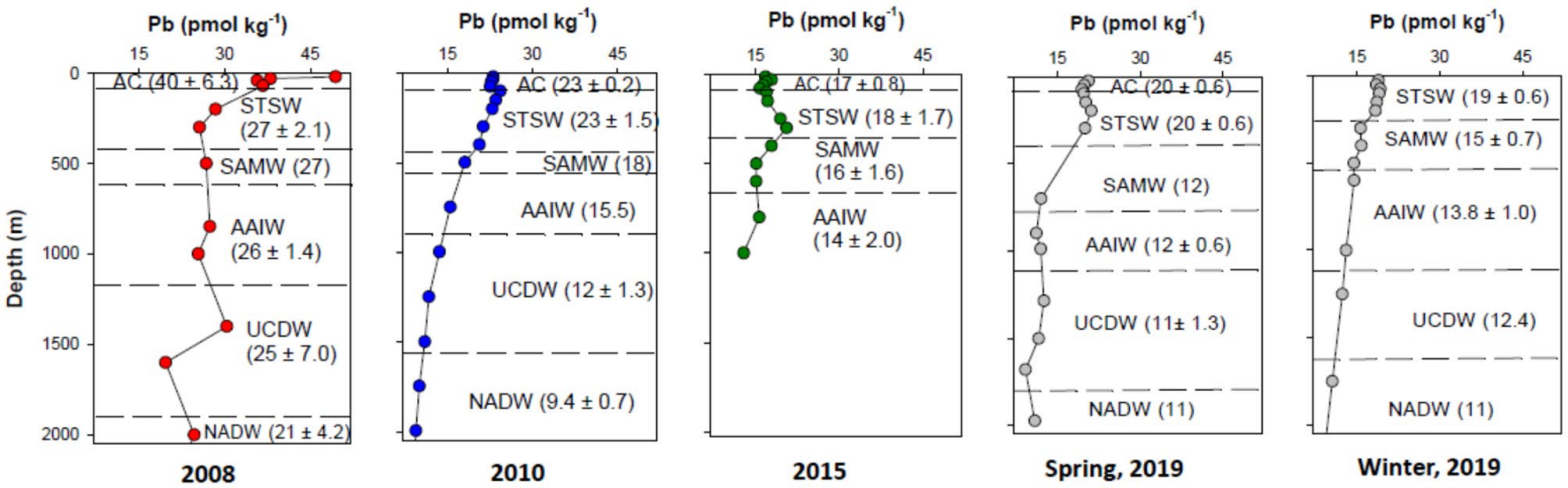
Vertical dPb distribution over time at station GT10 in the Cape Basin. Different water masses encountered with depth are identified and their respective average dPb ± 1σ concentrations are given in brackets. Water masses were identified based on Fig. 4. Data for the years 2008 and 2010 were previously published^30,31^. AC water mass is not evident in 2019 due to its minimum contribution in winter (discussed in section "Seasonal variation of AC-derived Pb to the Cape Basin").

In 2008, dPb was higher in all identified water masses compared to measurements in subsequent years (Fig. 5). Furthermore, in 2008, the average dPb in the AC-derived water mass (40 ± 6.3 pmol kg^−1^, n = 4) was nearly twice as high compared to the average dPb in the underlying STSW (27 ± 2.0 pmol kg^−1^, n = 2) with concentrations only slightly varying in deeper water masses. However, in 2010 and thereafter, the upper two water masses had comparable dPb concentrations. For instance, in 2019 (Spring), measured dPb was 20 ± 1.6 pmol kg^−1^ both in the STSW (n = 3) and AC (n = 4). As the Cape Basin is least influenced by AC during winter 2019 (Figs. 2 and 3), the AC-derived water was not evident in the winter of 2019.

On a decadal scale, the highest variability in the southeast Cape Basin was observed in the AC-derived waters with a sharp decline in dPb (from 40 ± 6.3 to 23 ± 0.2 pmol kg^−1^) observed between 2008 and 2010. Given that dPb concentrations in the surface ocean are primarily contributed through wind-driven atmospheric Pb from the continents, it is reasonable to assume that the observed drop in dPb in the Cape Basin could be related to the switch from the use of leaded to unleaded petroleum in South Africa in 2008^29^. Similarly, the marginal rise in dPb in the AC-derived water between 2015 and 2019 (Fig. 5) does not rule out the possible effect of increased use of Pb-bearing raw materials in South Africa.

### Aerosol mediated Pb in the Cape Basin

Before leaded petroleum was banned in South Africa in 2008, it contributed ca. 70% to the atmospheric Pb^46^. The subsequent lower Pb emissions (ca. 3000 ton/year^21^) were reflected in the Pb content of South African aerosols sampled from the Northwest province, where Pb/Al values dropped from ca. 110 × 10^–3^ to 40 × 10^–3^ between 2006 and 2010^47–51^ (Supplementary Fig. S4). That is, the decreased dPb concentration in the AC-derived water of the Cape Basin during 2008–2010 (Fig. 5) conforms to the decline in South African aerosol Pb. This would suggest that South African aerosols contribute a major fraction of Pb in the Cape Basin. However, given that the aerosols generated in Southern Africa are not transported westward to the Cape Basin (Supplementary Fig. S5), we posit that the transference of the signal is indirect. In 2019, Pb data of the aerosols collected in coastal oceans around South Africa show three times higher Pb concentrations and Pb/Al ratios on the southeast coast compared to the southwest coast^52^. The aerosols that accumulated airborne particles from the continent and deposited along the AC water on the east coast, display the highest levels of Pb (230 pg kg^−1^) and Pb/Al (8.1 × 10^–3^) (Supplementary Fig. S5). This, along with the dPb distribution in the Southwest Indian Ocean, which shows elevated dPb distributions close to the South African coast, confirms a considerable Pb input from South Africa to the Agulhas water along the southeast coast^35^.

Despite the phasing out of leaded petroleum, we observed a marginal increase between 2015 and 2019. In recent years developing nations such as South Africa and South Asian countries have increased the use of other Pb-emitting raw materials (coal, ore, unleaded petroleum)^19,21^. Consequently, Pb emissions have increased, but not to the same extent as when leaded petroleum was used^21^. While assessing the use of Pb-emitting raw materials in South Africa, we found a few instances which support increasing Pb emissions between 2015 and 2019. For example, the selected coal-fired power plants, located close to the East Coast, showed a four- to five-fold rise in particulate matter (PM) emissions because of an increased requirement for power generation^53,54^. Among petroleum products, the use of bitumen increased significantly as observed by the expansion of roads from 335,000 to 747,000 km between 2006 and 2017^55^. A 30% increase in total vehicles during 2015–2017^56^ further supports increasing Pb emissions from vehicles and roads. The impacts of these activities are corroborated by the temporal Pb/Al data of South African aerosols, which show an increase during 2015–2016 (Supplementary Fig. S4). That is, the marginal rise in AC-derived dPb in the Cape Basin between 2015 and 2019 was likely caused by the increased Pb in the South African aerosols.

The relative contribution of Pb from South African aerosols and dPb-enriched North Indian Ocean waters remains unclear due to a lack of dPb and Pb isotope data from the Southwest Indian Ocean. However, the dPb distribution in the Indian Oceans shows a difference in the dPb signature between the Southwest Indian Ocean (AC) waters, and the North and Central Indian Ocean waters (Supplementary Fig. S6). As a result, it appears that atmospheric Pb from Asia is not the primary source of Pb in the Southwest Indian Ocean. Based on the consistent temporal change in the Pb emissions from South Africa and dPb concentrations in the Cape Basin, we further posit that in the Cape Basin, a significant portion of the dPb was of atmospheric origin. However, atmospheric Pb from South Africa was first precipitated in the West Indian Ocean, then transported to the South Atlantic through the AC.

## Conclusions

Dissolved Pb concentrations measured in biologically productive waters of Southeast Cape Basin are generally higher than the remaining South Atlantic sector and are a function of mixing between STSW with AC waters. Previously hypothesized exchange of dPb between SW Indian and South Atlantic Ocean via AC waters was thus quantified using a mixing diagram and dPb data measured between 2008 and 2019. From the surface to the mixed layer depth (varied from 50 to 140 m) in the Cape Basin, the water is dominated by the AC, accounting for 30 and 90% of the measured dPb with the higher-end flux contribution with the surface (< 5 m) waters. The transfer flux varied, increasing from winter to summer according to the variation in the strength of AC, which impacts the water input into the South Atlantic. By comparing the decadal scale dPb measurements (2008–2019) with Pb transport and variability in South African aerosols, it appears that South African dust is an indirect, but significant source of Pb. That is, atmospheric Pb from South Africa is first precipitated in the West Indian Ocean and is then transported to Cape Basin mediated via AC.

This study ascertains the role of AC in bringing dPb to the Cape Basin where any significant direct sources of Pb otherwise are lacking. South Africa, like other developing nations (e.g., Southeast Asian countries^19^), is vulnerable to increased Pb emissions in future years due to its dependence on coal energy and related economic growth. This, along with an increase in Agulhas leakage in recent decades^57,58^, strengthens the likelihood of future increases in the AC-derived dPb in a changing climate scenario. Thus, to protect the ecologically sensitive and commercially rich fishing grounds of Cape Basin, this study highlights the need for long-term regulation of atmospheric Pb emissions from Southern African Countries.

## Methods

### Sampling and analyses

In the South Atlantic sector close to the southwest coast of South Africa, surface (< 5 m depth) and deep-water samples were collected, while along the southeast coast of South Africa, only surface waters were collected. (Fig. 1). Sampling was performed on-board the R/V SA Agulhas II in January 2015 (South African National Antarctic Expedition, SANAE 54) and July–November 2019 (Southern oCean seAsonaL Experiment, SCALE) following ‘trace metal clean’ GEOTRACES sampling protocols^59^. Surface seawater (< 5 m) was collected using a towed-fish sampling module deployed off the side of the ship at a speed of less than 10 knots. The epoxy coated towed-fish included a PFA Teflon® nozzle to accommodate inside an acid-clean polypropylene® plastic tubing for pumping uncontaminated water directly to a Class 100 clean container lab. Water was pumped using a PTFE Teflon® diaphragm pump (Vegapumps® CE) connected to an air compressor (Lab-air® Quiet Clean Oil-free). Surface sampling was performed in winter (in the Indian Ocean sector) and spring (South Atlantic sector) (Fig. 1). The vertical profiles were sampled using clean Teflon® coated 12 l GO FLO bottles (General Oceanics) mounted on a GEOTRACES-compliant titanium CTD rosette, as described previously^60,61^. The vertical profiles at CTD stations GT10 and GT9 were sampled in both the winter and spring of 2019 (Fig. 1). For both surface and vertical sampling, seawater was filtered through 0.2 µm Sartobran® capsule filters and collected in 125 ml acid-cleaned low-density polyethylene (LDPE) bottles to measure the dissolved fraction of Pb. Each sample was acidified (pH ~ 1.7) by adding 250 µL 30% HCl (Merck® ultrapur). A set of unfiltered samples was separately collected in 2015, considered as the total dissolvable fraction.

Measurement of dissolved metals including dPb was performed using the established analytical setup in the TracEx laboratory, University of Stellenbosch, South Africa^62^. The analytical setup comprised a commercially available preconcentration unit (ESI® seaFAST S3) and a single quadrupole ICPMS (Agilent® Mass hunter 7900). Samples were analysed using either an offline setup (2015) or an online setup (2019). The measurement accuracy was determined by analysing certified (e.g., NASS7), GEOTRACES, and in-house reference seawater (Table 1). Based on the duplicate analysis of seawater samples, the analytical precision of dPb was estimated to be better than 5%. Based on the analyses of the procedural blank (1% HCl, Merck® ultrapur), the limit of detection (3 × 1σ_blank_) was estimated to be 0.08 pmol kg^-1^.

**Table 1:** The results of the accuracy tests.

Standard	n	Certified/consensus/calibrated concentration (pmol kg^−1^)	Measured concentration (pmol kg^−1^)
NAAS7	6	12 ± 3.86	11 ± 0.33
GSC	5	39 ± 4	41 ± 5
GSP	5	62 ± 5	62 ± 8
WISOS-TM1	> 22	15 ± 0.5	14 ± 1.2
SCALE-GT2B_calibrated_	> 25	9.4 ± 0.8	9.2 ± 0.8
SCALE-GT8_calibrated_	> 27	10.7 ± 1.0	10.2 ± 1.2

The previously published dPb data^30,31^ of vertical profiles used in this study were sampled during the multidisciplinary MD 166 BONUSGoodHope cruise (R/V Marion Dufresne II; Feb to Mar 2008) and UK GEOTRACES GA10 cruise (RRS Discovery; Oct-Nov 2010). The published dPb data^28,35^ for the surface waters (< 5 m) were collected during the UK GEOTRACES GA10 cruise (RRS Discovery; Oct-Nov 2010) in the South Atlantic sector and the Malaspina 2010 Circumnavigation Expedition (R/V Hesperides; Jan-Feb 2011) in the Indian Ocean sector. For each study, trace metal clean sampling and analyses were performed. For more information, please refer to the original papers.

### Estimation of spiciness

Spiciness (τ) is a function of absolute salinity (S_A_), conservative temperature (T_c_), and pressure (p)^37,38^. First, the S_A_ and C_T_ were derived from S_p_ and pT, respectively.

The derivation of S_A_, T_c_, and τ was performed in Matlab using the Gibbs SeaWater (GSW) Oceanographic Toolbox of TEOS-10 (http://www.TEOS-10.org).

### Preparation of mixing diagram

The concept of mixing diagram was followed from the conventional mixing diagram of Pb using ^206^Pb/^207^Pb vs. 1/dPb^7,35^. The equations used to draw a mixing diagram of dPb are based on the following equations:

1 $${\frac{{Pb}^{206}}{{Pb}^{207}}}_{mix}=\frac{{dPb}_{em1}\times {\frac{{Pb}^{206}}{{Pb}^{207}}}_{em1}\times {f}_{em1}+{dPb}_{em2}\times {\frac{{Pb}^{206}}{{Pb}^{207}}}_{em2}\times {f}_{em2}}{{dPb}_{em1}\times {f}_{em1}+{dPb}_{em2}\times {f}_{em2}}$$

2 $${{dPb}_{mix}=dPb}_{em1}\times {f}_{em1}+{dPb}_{em2}\times {f}_{em2}$$

where f represents the fractional contribution and subscript *em* stands for the end-members. The isotopic mixing of Pb produces a nonlinear trend with salinity, while the mixing of dPb concentration forms a linear trend with salinity^13,36^. Therefore, ^206^Pb/^207^Pb vs. 1/dPb diagram produces a linear mixing trendline (Supplementary Fig. 2). In this study, we construct the mixing diagram, spiciness (τ) vs. 1/Pb, based on the following equations:

3 $${{\tau }_{mix}=\tau }_{em1}\times {f}_{em1}+{\tau }_{em2}\times {f}_{em2}$$

4 $${{dPb}_{mix}=dPb}_{em1}\times {f}_{em1}+{dPb}_{em2}\times {f}_{em2}$$

As we use 1/Pb instead of Pb, the plot of τ vs. 1/Pb produces a non-linear mixing curve between the two end-members (Fig. 2c, f).

The end-member values of dPb and τ were determined by averaging the samples collected from the regions characterizing the water mass (Fig. 2a, b, d, e). For example, the dPb_AC_ and τ_AC_ represent the estimated values based on the samples collected along the pathway of AC on the east coast of South Africa (Table 2). The STSW variables were estimated using the samples from the south of the Cape Basin in the South Atlantic sector. Due to the unavailability of pT and S_p_ of the AC in 2010^35^, we use the τ_AC_ of 2019 to draw the mixing curve in 2010 (Fig. 2f). The standard deviation associated with the dPb_AC_ is derived based on the analytical precision of dPb (< 5%).

**Table 2:** End member values of variables used in the mixing calculations.

Source water	Year	dPb (pmol^−1^ kg)	τ
AC	2019	29 ± 2.5 (n = 10)	3.99 ± 0.15 (n = 10)
STSW	2019	15 ± 2.4 (n = 10)	2.51 ± 0.28 (n = 10)
AC^35^	2010	29 ± 1.4 (n = 1)	–
STSW^28^	2010	16 ± 2.3 (n = 3)	2.25 ± 0.21 (n = 3)

–, data unavailable.

When we use Pb instead of using 1/Pb in the mixing diagram (τ vs. Pb), it produces linear mixing line, however, the outcome remains the same (Supplementary Fig. S7).

### Identification of the frontal position

STF is a boundary that prevents subtropical surface water (STSW) from drifting further south and subantarctic surface water (SASW) from drifting further north. STF is defined by a steep rise of isohalines lines from 34.6 to 35.1^63–65^. For the identification of STF during winter and spring cruises in 2019, we use the vertical distribution of salinity along the South Atlantic sectors, following approximately the GEOTRACES GIPY04 transect^66^.

### Identification of water masses

The different water masses in the study region were identified using pT and Sp. The end member values for the water masses were selected using the published datasets^67–78^, compiled in Table 3. The traditional pT-S_p_ bidirectional plot with potential density contours was used to identify different water masses in the vertical profile (Fig. 3).

**Table 3:** In situ properties of different water masses.

Water mass	pT (°C)	S_p_	Sources
AC	20.08 ± 0.60	35.42 ± 0.01	This study
STSW	15.05 ± 2.55	35.25 ± 0.32	^67–70,72^
SASW	7.83 ± 1.44	34.10 ± 0.14	^70,75^
AASW	0.34 ± 2.64	34.04 ± 0.25	^68,69,71,76,78^
SAMW	10.33 ± 0.01	34.58 ± 0.10	^68,69,71^
AAIW	3.99 ± 0.64	34.14 ± 0.20	^68–76^
UCDW	3.09 ± 1.10	34.46 ± 0.18	^69,75,78^
NADW	3.30 ± 1.29	34.95 ± 0.06	^68,73^
LCDW	1.50 ± 0.72	34.73 ± 0.03	^68,69,71,72^
AABW	− 0.49 ± 0.61	34.59 ± 0.14	^68,69,71,73,75^

AC, Agulhas water; STSW, subtropical surface water; SASW, Subantarctic surface water; AASW, Antarctic surface water; SAMW, Subantarctic mode water; AAIW, Antarctic intermediate water; UCDW, upper circumpolar deep water; NADW, North Atlantic deep water; LCDW, lower circumpolar deep water; AABW, Antarctic bottom water.

## Supplementary Information

Supplementary Information.

## Data Availability

The previously published data (pT, S_p_ and dissolved Pb) are available in the Geotraces Intermediate data product 2017^79^. The SLA and geostrophic current datasets for Fig. 4 were obtained from the Copernicus Climate Change Service (C3S) ( https://cds.climate.copernicus.eu/cdsapp#!/dataset/10.24381/cds.4c328c78?tab=overview). SLA is the gridded merged product of several altimeter observations: e.g., TOPEX/Poseidon, Jason—1, Jason—2, Jason—3, ERS—1, ERS—2, etc. And geostrophic velocity is a derived product from SLA observations.
