# Supplementary material for: Exchange of Pb from Indian to Atlantic Ocean is driven by Agulhas current and atmospheric Pb input from South Africa
Source: Sci Rep. 2023 Apr 4;13:5465. doi: 10.1038/s41598-023-32613-5 (PMC10073131; doi:10.1038/s41598-023-32613-5)
Supplement: Supplementary file 1 — Supplementary Information. [file 41598_2023_32613_MOESM1_ESM.pdf]

Supplementary information

Exchange of Pb from Indian to Atlantic Ocean is driven by Agulhas Current and atmospheric Pb input from South Africa

Saumik Samanta[1]*, Ryan Cloete[1], Subhra Prakash Dey[2, 3], Jan-Lukas Menzel Barraqueta[1], Jean C Loock[1], Jan-Olaf Meynecke[4], Jasper de Bie[4], Marcello Vichi[2], Alakendra N Roychoudhury[1]*

[1]Centre for Trace Metal and Experimental Biogeochemistry (TracEx), Department of Earth Sciences, Stellenbosch University, Stellenbosch, 7600, South Africa
[2]Department of Oceanography, University of Cape Town, Rondebosch, 7701, South Africa
[3]CSIR - National Institute of Oceanography, Dona Paula, Goa 403004, India
[4]Coastal and Marine Research Centre, Griffith University, Gold Coast, Qld, Australia
*Corresponding author
Saumik Samanta (saumiksamanta@gmail.com; 22505709@sun.ac.za)
Alakendra N Roychoudhury (roy@sun.ac.za)

Supplementary text

ST1. Quantitative change in dPb concentrations in the study area

Processes such as the low residence time of dPb in surface seawater, riverine input to the coastal oceans, and the effect of evaporation-precipitation may change the dPb concentration in the surface seawater making it act non-conservatively. Our calculations below show minimal impact on dPb from these processes in the study area thereby allowing the use of dPb in mixing diagrams. First, using a velocity of AC (2.0±1.0 m/sec)[1,2] in upper 100 m, and an approximate oceanographic distance between East London and Cape Basin (2000 km; Fig. 1 main text); the travel time for Agulhas water from the Indian Ocean to Cape basin is estimated to be 12±6 days. The resulting travel time of AC is considerably shorter compared to the residence time of dPb in the surface ocean of the studied region (from 1 to 10 y)[3]. Second, based on annual precipitation of 600±200 mm (average of annual precipitation in Cape Town and East London) and the total surface area of the studied region (9.7×10^5 km^2; area within 32°-36°S and 10°-30°E), the estimated water volume due to precipitation accounts for ca. 0.02% of the annual interoceanic water transfer through Agulhas leakage (12.2 Sv)[4]. Using elevated dPb concentrations for the rainwater harvested from a roof in KwaZulu-Natal, a coastal city close to our study area (ca. 100 pmol kg^{-1})[5], precipitation could add up to only 0.02 pmol kg^{-1} in the surface seawater, which is below the detection limit of dPb (see Methods, main text). Similarly, a significant change in dPb concentration due to evaporation is also unexcepted. Third, there are a few rainwater-fed rivers that drain from South Africa to the Southwest Indian Ocean. However, the discharge from these rivers is relatively small compared to the major river systems of the world. For instance, using the annual runoff of the Breede river (1784×10^6 m^3/sec), a South African river with the fourth-highest water discharge, and the average dPb concentration of the African rivers (190 pmol kg^{-1})[6] , it is estimated that the riverine dPb discharge could increase the dPb concentration in the AC water by 0.001 pmol kg^{-1}. The above observations support using dPb while assessing the mixing of the AC and STSW in the Cape Basin.

Supplementary Figures

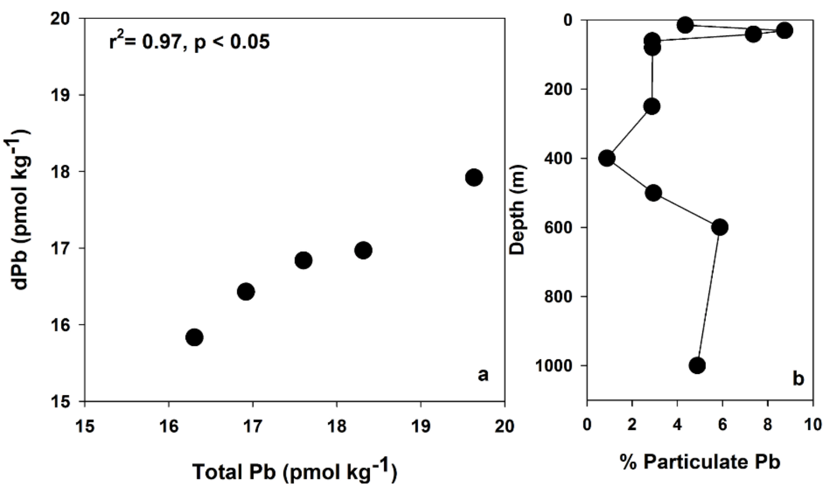

Figure S1. a. Distribution of dissolved and total Pb in the upper 150 m of the Cape Basin. b. vertical distribution of % particulate Pb (calculated based on dissolved and total Pb data) in the Cape Basin.

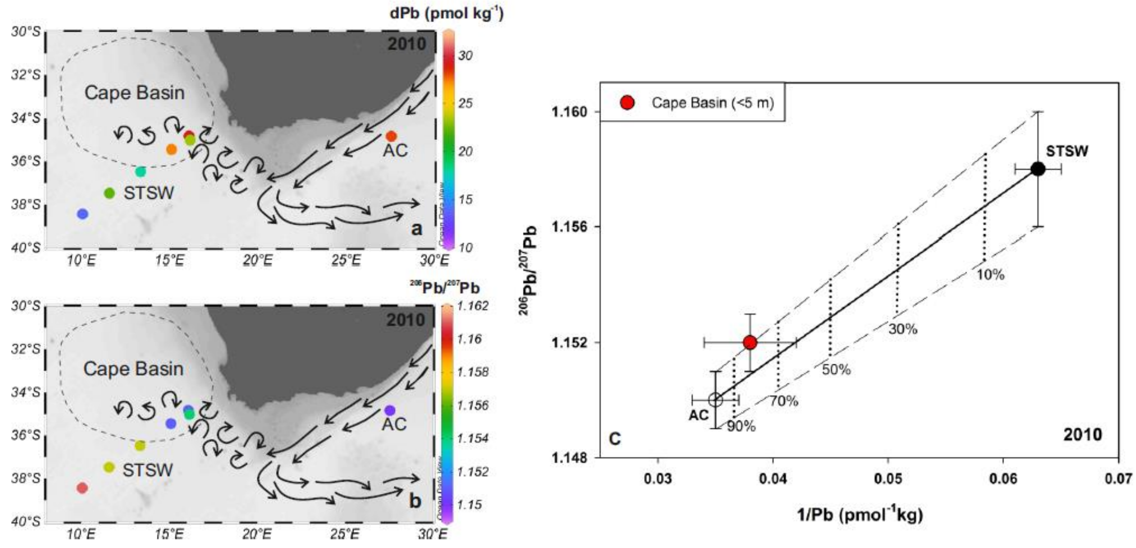

Figure S2. Distribution of dPb (a) and Pb isotopic composition (b) in the Subtropical Ocean around South Africa in 2010[7–9] and the corresponding mixing diagram (c). The end member ($^{206}Pb/^{207}Pb$ and Pb) for STSW (black filled circles) and AC (open circle) in the mixing diagram were calculated from the average and standard deviation of the samples collected in different oceanic regions representing the respective water masses shown in the left panels. The bold black line in the mixing diagram indicates the mixing between absolute values of the two end-members. The black dashed lines also indicate the mixing considering the standard deviation associated with the absolute values of Pb isotopic composition. The dotted lines represent the contribution of AC water constructed considering the range of Pb isotopic composition. The end member for AC was constrained based on single available datapoint and the standard deviation was calculated based on the measurement precisions.

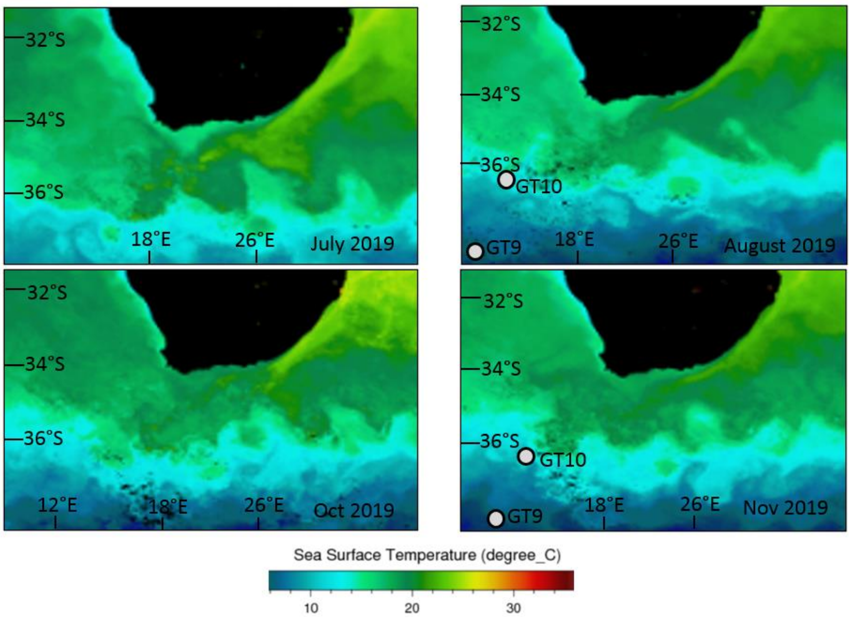

Figure S3. Monthly average sea surface temperature (SST) map (MODIS-Terra SST, 11 μ daytime; https://oceancolor.gsfc.nasa.gov/) in the study area during winter (upper panels) and spring (lower panels), 2019. The map shows a minimal change in SST at GT10 station between August and November 2019.

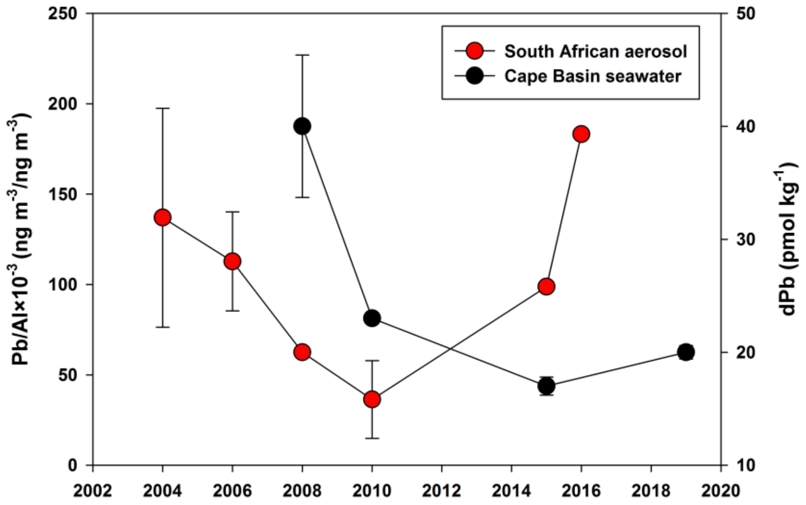

Figure S4. Pb/Al ratios of aerosols sampled from South Africa [10–14]. The declining Al-normalized Pb concentration during 2006-2010 follows the change in Pb emissions from South Africa. An increase in later years corresponds to an increased use of Pb containing raw materials in various industrial streams. The dPb concentrations in the Cape Basin also follow the similar pattern.

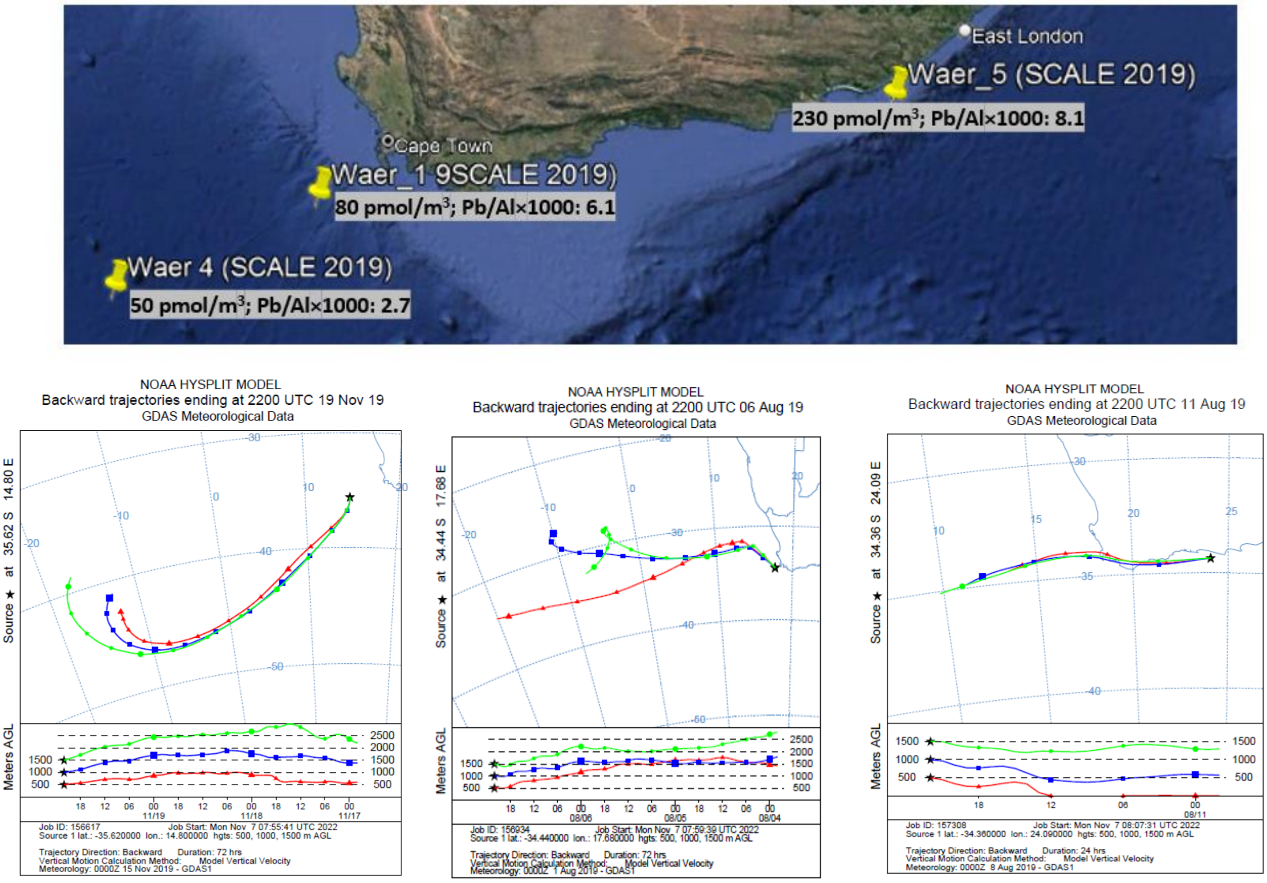

Figure S5. Upper panel: aerosols collected from the southeast coast showing enriched Pb concentrations and higher Pb/Al ratios compared to the southwest coast[15]. Lower panels: The air mass trajectories show the aerosols from the southeast coast accumulate dust from South Africa. Air mass trajectory was made using NOAA hysplit backward trajectory model[16].

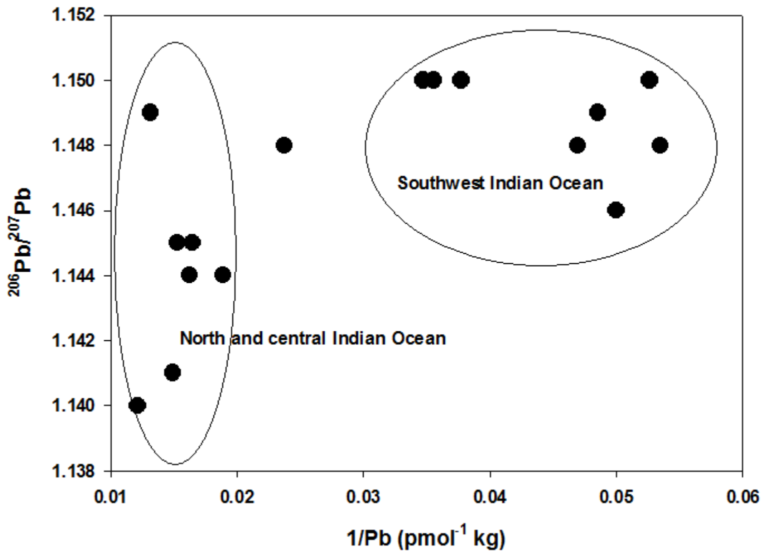

Figure S6. dPb distributions in the Indian Ocean [9,17,18]. The Southwest Indian Ocean waters show different Pb signature compared to the North and Central Indian Ocean waters.

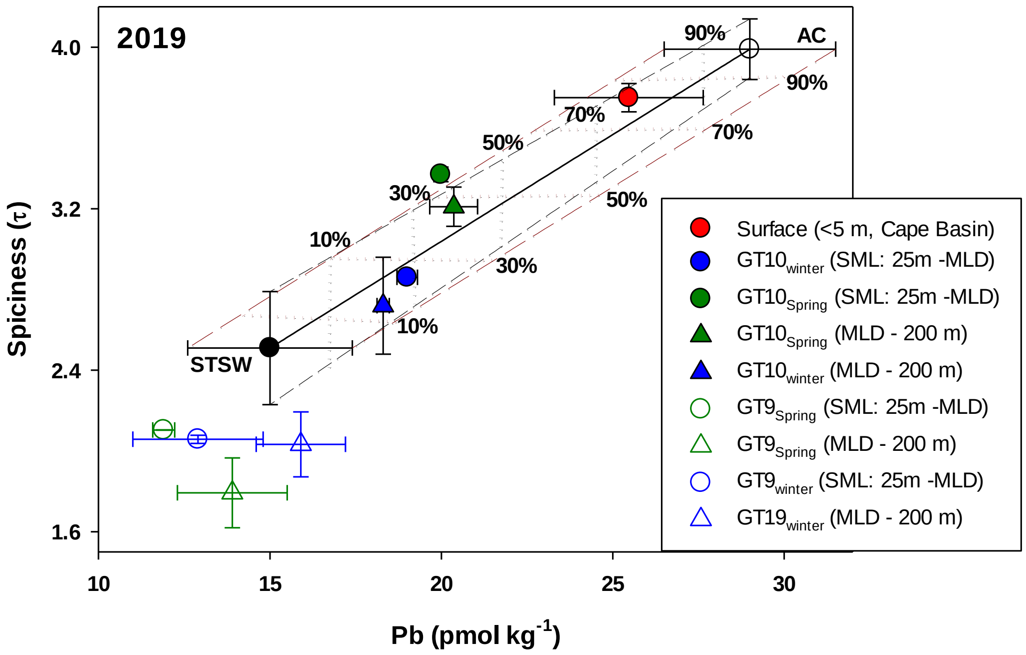

Figure S7. Mixing diagram based on τ and dPb concentrations. The end members (τ and Pb) for AC (open circle) and STSW (black-filled circle) in the mixing plots were calculated from the average and standard deviation of the samples collected in different oceanic regions representing the respective water masses shown in Figure 2a and 2b (main text). The bold black line in the mixing diagram (c and f) indicates the mixing between absolute values of the two end-members. The dashed lines also indicate the mixing considering the standard deviation associated with the absolute values of the end-members (black corresponds to τ and brown corresponds Pb). The dotted lines represent the contribution of AC water constructed considering both the range of τ (black) and Pb (brown).

Table S1. Potential temperature (pT), practical salinity (Sp) and dPb concentrations of the surface water (<5 m)

Sample No.	Date of sampling	Latitude	Longitude	S_p	pT (°C)	dPb (pmol kg^{-1})
FISH 23-W	06.08.2019	-34.55	22.71	35.41	17.07	-
FISH 24-W	06.08.2019	-34.51	22.99	35.43	17.42	24
FISH 25-W	06.08.2019	-34.47	23.25	35.44	17.60	24
FISH 26-W	06.08.2019	-34.42	23.55	35.45	17.67	25
FISH 27-W	06.08.2019	-34.39	23.83	35.45	18.11	21
FISH 28-W	06.08.2019	-34.37	24.13	35.47	18.19	28
FISH 29-W	06.08.2019	-34.35	24.44	35.44	18.64	25
FISH 30-W	06.08.2019	-34.32	24.69	35.45	18.57	26
FISH 31-W	06.08.2019	-34.28	25.12	-	-	28
FISH 32-W	06.08.2019	-34.24	25.56	-	-	28
FISH 33-W	06.08.2019	-	-	-	-	-
FISH 34-W	07.08.2020	-34.19	25.90	-	-	24
FISH 35-W	07.08.2020	-	-	-	-	-
FISH 36-W	07.08.2020	-34.10	26.14	35.44	21.25	31
FISH 37-W	07.08.2020	-33.99	26.42	35.43	20.15	30
FISH 38-W	07.08.2020	-33.86	26.67	35.43	20.63	27
FISH 39-W	07.08.2020	-33.75	26.93	35.4	20.79	26
FISH 40-W	07.08.2020	-33.65	27.19	35.42	21.09	25
FISH 41-W	07.08.2020	-33.47	27.43	35.41	19.78	29
FISH 42-W	07.08.2020	-33.32	27.61	35.41	21.20	32
FISH 43-W	07.08.2020	-33.16	27.81	35.40	19.98	27
FISH 44-W	07.08.2020	-33.07	27.93	35.42	20.82	31
FISH 1-S	13.10.2019	-36.05	13.99	35.56	19.69	23
FISH 2-S	13.10.2019	-36.22	13.53	35.53	18.85	23
FISH 3-S	13.10.2019	-36.48	13.18	35.32	17.09	16
FISH 4-S	13.10.2019	-37.23	12.69	35.13	15.36	17
FISH 5-S	14.10.2019	-37.59	12.45	35.39	15.92	17
FISH 6-S	14.10.2019	-37.95	12.22	34.94	14.07	11
FISH 7-S	14.10.2019	-38.14	12.10	34.77	13.52	-
FISH 8-S	14.10.2019	-38.33	11.97	35.11	14.71	14
FISH 9-S	14.10.2019	-38.69	11.73	35.19	15.02	15
FISH 10-S	14.10.2019	-39.01	11.50	34.99	13.75	12
FISH 11-S	14.10.2019	-39.30	11.29	34.79	12.63	13
FISH 23-S	18.11.2019	-36.10	13.74	35.56	19.35	25
FISH 24-S	18.11.2019	-36.02	13.91	35.56	19.24	24
FISH 25-S	18.11.2019	-35.94	14.08	35.56	19.18	25
FISH 26-S	18.11.2019	-35.86	14.25	35.56	19.29	24
FISH 27-S	18.11.2019	-35.78	14.43	35.56	18.98	24
FISH 28-S	18.11.2019	-35.71	14.60	35.56	19.13	25
FISH 29-S	18.11.2019	-35.63	14.77	35.56	19.24	23
FISH 30-S	19.11.2019	-35.55	14.94	35.56	19.12	22
FISH 31-S	19.11.2019	-35.48	15.10	35.55	19.37	22
FISH 32-S	19.11.2019	-35.40	15.27	35.56	19.35	26

FISH 33-S	19.11.2019	-35.32	15.43	35.56	19.48	24
FISH 34-S	19.11.2019	-35.24	15.61	35.54	19.71	30
FISH 35-S	19.11.2019	-35.15	15.80	35.54	19.73	30
FISH 36-S	19.11.2019	-35.08	15.95	35.54	19.59	28
FISH 37-S	19.11.2019	-35.01	16.11	35.53	19.85	29

- Data not available

Table S2. Water column distribution of pT, S_p and dPb in the vertical profiles

**Sample No.**	**Date of sampling**	**Latitude**	**Longitude**	**Depth (m)**	$\mathbf{S_p}$	**pT (°C)**	**dPb (pmol kg^{-1})**
TM4	15.02.2015	-36.0	13.3	15	35.39	19.69	17
				31	35.39	19.66	18
				41	35.39	19.66	17
				60	35.26	17.14	16
				80	35.15	15.26	16
				100	35.27	14.83	17
				150	35.04	13.25	17
				250	35.13	12.81	19
				301	35.05	12.06	21
				400	34.86	10.68	18
				501	34.65	8.93	15
				600	34.52	7.56	15
				801	34.29	4.76	16
				1,000	34.36	3.91	13
GT-10	18.11.2019	-36.3	13.3	25	35.61	17.79	21
				49	35.61	17.78	20
				73	35.59	17.59	19
				97	35.59	17.48	20
				148	35.57	17.21	20
				197	35.54	16.84	21
				297	35.44	15.37	20
				494	35.14	12.60	12
				701	34.63	8.68	12
				989	34.39	5.65	12
				1,285	34.38	3.47	13
				1,501	34.57	3.34	12
				1,680	34.65	2.85	9.2
				1,973	34.74	2.70	11
GT-10	05.08.2019	-36.3	13.3	26	35.48	16.02	19
				50	35.48	16.02	19
				75	35.48	16.02	19
				100	35.48	15.94	19
				125	35.48	15.92	-

				150	35.43	15.61	19
				200	35.29	14.18	18
				301	35.07	12.57	16
				400	34.95	11.33	16
				501	34.76	9.81	14
				600	34.60	8.40	15
				1,002	34.34	4.28	13
				1,251	34.47	3.43	12
				1,750	34.70	2.73	11
				2,251	34.81	2.50	8.8
				2,750	34.84	2.23	7.8
				3,501	34.83	1.74	7.7
				4,252	34.75	0.88	5.8
GT-9	17.11.2019	-38.4	11.5	15	35.05	14.05	12
				26	35.05	14.04	12
				34	35.06	13.76	12
				52	34.96	13.14	13
				74	35.01	13.24	17
				100	34.92	12.80	14
				151	34.89	12.20	13
				200	34.97	12.01	13
				401	34.68	9.15	15
				601	34.38	6.06	12
				800	34.24	4.18	12
				999	34.30	3.43	10
				1,301	34.54	3.23	8.6
				1,699	34.71	2.75	10
				1,999	34.81	2.72	12
GT-9	04.08.2019	-38.4	11.5	26	35.13	14.23	14
				50	35.06	13.71	14
				75	35.08	13.69	14
				100	35.09	13.63	15
				125	35.22	13.78	18
				150	35.21	13.54	16
				200	35.06	12.59	15
				300	34.97	11.50	15
				400	34.83	10.32	15
				500	34.67	8.98	-
				750	34.30	5.18	-
				1,000	34.31	3.76	-
				1,250	34.44	3.09	-
				1,500	34.58	2.80	-
				1,750	34.71	2.69	-
				2,250	34.82	2.50	-
				2,750	34.84	2.22	-

3,500	34.82	1.74	-
4,189	34.76	0.96	-
4,667	34.74	0.79	-

-Data unavailable

References

1. Flemming, B. W. Factors controlling shelf sediment dispersal along the southeast African continental margin. *Mar Geol* **42**, 259–277 (1981).

2. Bryden, H. L., Beal, L. M. & Duncan, L. M. Structure and Transport of the Agulhas Current and Its Temporal Variability. *J Oceanogr* **61**, 479–492 (2005).

3. Henderson, G. M. & Maier-Reimer, E. Advection and removal of 210Pb and stable Pb isotopes in the oceans: A general circulation model study. *Geochim Cosmochim Acta* **66**, 257–272 (2002).

4. Durgadoo, J. v., Rühs, S., Biastoch, A. & Böning, C. W. B. Indian Ocean sources of Agulhas leakage. *J Geophys Res Oceans* **122**, 3481–3499 (2017).

5. Selala, M., Thenga, H., Jewitt, G. & Chaplot, V. Comparison of the chemical quality of rainwater harvested from roof and surface run-off systems. *Water SA* **44**, (2018).

6. Gaillardet, J., Viers, J. & Dupré, B. Trace Elements in River Waters. in *Treatise on Geochemistry* 225–272 (Elsevier, 2003). doi:10.1016/B0-08-043751-6/05165-3.

7. Paul, M. *et al.* Tracing the Agulhas leakage with lead isotopes. *Geophys Res Lett* **42**, 8515–8521 (2015).

8. Schlosser, C., Karstensen, J. & Woodward, E. M. S. Distribution of dissolved and leachable particulate Pb in the water column along the GEOTRACES section GA10 in the South Atlantic. *Deep Sea Res 1 Oceanogr Res Pap* **148**, 132–142 (2019).

9. Pinedo-González, P., West, A. J., Tovar-Sanchez, A., Duarte, C. M. & Sañudo-Wilhelmy, S. A. Concentration and isotopic composition of dissolved Pb in surface waters of the modern global ocean. *Geochim Cosmochim Acta* **235**, 41–54 (2018).

10. Venter, A. D. *et al.* Atmospheric trace metals measured at a regional background site (Welgegund) in South Africa. *Atmos Chem Phys* **17**, 4251–4263 (2017).

11. Segakweng, C. K. *et al.* Measurement report: Size-resolved chemical characterisation of aerosols in low-income urban settlements in South Africa 2. *Atmos Chem Phys* (2022) doi:10.5194/acp-2021-1026.

12. van Zyl Pieter G. *et al.* Assessment of atmospheric trace metals in the western Bushveld Igneous Complex, South Africa. *S Afr J Sci* **110**, 1–11 (2014).

13. Sachsenberg, S., Klenke, T., Krumbein, W. E. & Zeeck, E. A back-extraction procedure for the dithiocarbamate solvent extraction method . Rapid determination of metals in seawater matrices. 163–164 (1992).

14. Kgabi Ed, N. A. *MONITORING THE LEVELS OF TOXIC METALS OF ATMOSPHERIC PARTICULATE MATTER IN THE RUSTENBURG DISTRICT*. http://repository.nwu.ac.za/bitstream/handle/10394/1504/kgabi_nnenesia.pdf;sequence=1 (2006).

15. Kangueehi, K. I. SOUTHERN AFRICAN DUST CHARACTERISTICS AND POTENTIAL IMPACTS ON THE SURROUNDING OCEANS. (Stellenbosch University, 2021).

16. Draxler, R. R. , & Rolph, G. D. ,. HYSPLIT (Hybrid Single-Particle Lagrangian Integrated Trajectory) Model. *NOAA, Air Resources Laboratory, Silver Spring* http://www.arl.noaa.gov/ready/hysplit4.html (2011).

17. Echegoyen, Y. *et al.* Recent distribution of lead in the Indian Ocean reflects the impact of regional emissions. *Proceedings of the National Academy of Sciences* **111**, 15328–15331 (2014).

18. Lee, J. M. *et al.* Impact of anthropogenic Pb and ocean circulation on the recent distribution of Pb isotopes in the Indian Ocean. *Geochim Cosmochim Acta* **170**, 126–144 (2015).
